# A Favorite Resort

**Published:** 1884-07

**Authors:** 


### THE FAVORITE SEASIDE RESORT.

Of all the seaside resorts on the New Jersey coast Atlantic City is probably the most patronized and the healthiest. In summer it is *the* place for cool air and fine bathing; in winter it is the great sanitarium, rivaling Florida in that respect. To those visiting Atlantic City who desire homœopathic treatment we can safely recommend Dr. J. H. Way (1905 Pacific Avenue), lately of West Chester, Pa.

